# Clinical Utility of a Commercial LAM-ELISA Assay for TB Diagnosis in HIV-Infected Patients Using Urine and Sputum Samples

**DOI:** 10.1371/journal.pone.0009848

**Published:** 2010-03-24

**Authors:** Keertan Dheda, Virginia Davids, Laura Lenders, Teri Roberts, Richard Meldau, Daphne Ling, Laurence Brunet, Richard van Zyl Smit, Jonathan Peter, Clare Green, Motasim Badri, Leonardo Sechi, Surendra Sharma, Michael Hoelscher, Rodney Dawson, Andrew Whitelaw, Jonathan Blackburn, Madhukar Pai, Alimuddin Zumla

**Affiliations:** 1 Lung Infection and Immunity Unit, Division of Pulmonology and Clinical Immunology and UCT Lung Institute, Department of Medicine, University of Cape Town, Cape Town, South Africa; 2 Institute of Infectious Diseases and Molecular Medicine, University of Cape Town, Cape Town, South Africa; 3 Clinical Research Support Unit, Department of Medicine, University of Cape Town, Cape Town, South Africa; 4 Centre for Infectious Diseases and International Health, Department of Infection, University College London Medical School, London, United Kingdom; 5 Division of Medical Biochemistry, University of Cape Town, Cape Town, South Africa; 6 Department of Epidemiology, Biostatistics and Occupational Health, McGill University, Montreal, Canada; 7 Dipartimento di Scienze Biomediche, University of Sardinia, Sassari, Italy; 8 Division of Pulmonary, Critical Care and Sleep Medicine, Department of Medicine, All India Institutes of Medical Sciences, New Delhi, India; 9 Division of Medical Microbiology, University of Cape Town, Cape Town, South Africa; 10 Department of Infectious Diseases and Tropical Medicine, Klinikum of the University of Munich, Munich, Germany; University of Stellenbosch, South Africa

## Abstract

**Background:**

The accurate diagnosis of TB in HIV-infected patients, particularly with advanced immunosuppression, is difficult. Recent studies indicate that a lipoarabinomannan (LAM) assay (Clearview-TB®-ELISA) may have some utility for the diagnosis of TB in HIV-infected patients; however, the precise subgroup that may benefit from this technology requires clarification. The utility of LAM in sputum samples has, hitherto, not been evaluated.

**Methods:**

LAM was measured in sputum and urine samples obtained from 500 consecutively recruited ambulant patients, with suspected TB, from 2 primary care clinics in South Africa. Culture positivity for *M. tuberculosis* was used as the reference standard for TB diagnosis.

**Results:**

Of 440 evaluable patients 120/387 (31%) were HIV-infected. Urine-LAM positivity was associated with HIV positivity (p = 0.007) and test sensitivity, although low, was significantly higher in HIV-infected compared to uninfected patients (21% versus 6%; p<0.001), and also in HIV-infected participants with a CD4 <200 versus >200 cells/mm^3^ (37% versus 0%; p = 0.003). Urine-LAM remained highly specific in all 3 subgroups (95%–100%). 25% of smear-negative but culture-positive HIV-infected patients with a CD4 <200 cells/mm^3^ were positive for urine-LAM. Sputum-LAM had good sensitivity (86%) but poor specificity (15%) likely due to test cross-reactivity with several mouth-residing organisms including *actinomycetes* and *nocardia* species.

**Conclusions:**

These preliminary data indicate that in a high burden primary care setting the diagnostic usefulness of urine-LAM is limited, as a rule-in test, to a specific patient subgroup i.e. smear-negative HIV-infected TB patients with a CD4 count <200 cells/mm^3^, who would otherwise have required further investigation. However, even in this group sensitivity was modest. Future and adequately powered studies in a primary care setting should now specifically target patients with suspected TB who have advanced HIV infection.

## Introduction

Tuberculosis (TB) kills almost two million people annually [1] and is out of control in Sub-Saharan Africa where up to 80% of TB patients may be co-infected with HIV [2]. Current tools for the diagnosis of TB are suboptimal and this contributes to poor control [3]. Delayed diagnosis facilitates disease transmission, increases healthcare costs, increases mortality, and causes greater lung damage resulting in chronic disability [4]. These drawbacks are exacerbated in HIV-infected patients, where the diagnostic ‘gap’ is the widest - smear-positivity in this sub-group is as low as 20% [5], and the clinical and the radiological features are often atypical [6]. Mycobacterial culture results are only available after several weeks, if at all, in resource-poor settings [7]. Newer technologies such as the T cell assays are not useful as rule-in tests for the diagnosis of active TB in adults [8], and molecular assays [7] are not widely available in high burden countries. The search for a rapid point-of-care (POC) test continues [9].

More recently a standardised and commercially available lipoarabinomannan (LAM) antigen-detection assay has been developed (Clearview® TB ELISA, Inverness Medical Innovations, USA; see http://www.clearview.com/tb_elisa.aspx), which now supersedes a pre-commercial prototype (MTB LAM ELISA Test®, Chemogen, Portland, USA) first tested in 2005 in Tanzania [10], and based on earlier developmental studies [11], [12]. This test option is attractive because urine is a sterile and easily obtainable biological fluid that can be assayed even in sputum scarce patients. Moreover, the assay has potential to be applied to other biological fluids e.g. cerebro-spinal fluid [13] and pleural fluid [14], and a user-friendly dipstick prototype of the test has been developed and is currently being validated as a POC test.

Recent studies have shown that urine LAM may have diagnostic value in HIV-infected [15], [16], [17], [18] but not in HIV-uninfected TB suspects [19]. However, studies that have stratified LAM outcomes by CD4 count are limited [15], [18] and there are no published data on CD4-stratified LAM-related outcomes in unselected TB suspects from a primary care setting. This information is crucial as most TB cases present to, and are diagnosed in primary care facilities in high burden settings. Thus, although it is known that LAM may be useful in HIV-infected patients [15], [18] the precise subgroups that may benefit from this test in a high burden primary care setting remain unknown. Another unresolved question, because there are currently no data, is whether the standardized urine Clearview® LAM ELISA assay has diagnostic utility when a sputum sample, in contrast to a urine sample, is used. Thus, the aims of the study were to determine the diagnostic accuracy of urine LAM in HIV-infected patients belonging to different CD4 T cell categories and to compare performance outcomes when using urine and sputum samples.

To address these questions, the Clearview® LAM ELISA was applied to concurrently collected urine and sputum samples. The assay is indicated for use with urine samples from HIV-infected subjects and thus the off-label evaluation of sputum specimens was exploratory.

## Methods

### Ethics Statement

Written informed consent was obtained from all participants and the study was approved by the University of Cape Town Human Research Ethics Committee.

### Study Sites and Population

500 consecutive ambulant self-reporting TB suspects (≥18 years of age) were recruited from 2 primary care clinics in Cape Town, South Africa (Langa Clinic is a primary health care facility in a disadvantaged Black African township with a high HIV prevalence, and Chapel Street is an inner-city clinic in a deprived area with a lower prevalence of HIV and a higher proportion of patients of mixed race).

All patients with symptoms suggestive of pulmonary TB were invited to participate in the study (any one of persistent fever, night sweats, cough of more than 2 weeks duration, hemoptysis, chest pain, loss of weight and/or appetite, and generalized fatigue). Detailed demographic information, clinical history and physical characteristics were recorded by a trained researcher on a standardized and validated case record form.

An HIV test was performed in all consenting study participants after appropriate counseling. Where a test result was positive, a CD4 count was also determined. Urine and a sputum samples were collected from each participant for LAM detection. A further 2 sputum samples were collected for routine fluorescence smear microscopy after centrifugation. These sputum samples were also cultured for *Mycobacterium tuberculosis* (BACTEC MGIT 960, BD Diagnostics, USA). Standard chest radiography, and a urine dipstick test (*Uri*CHECK 9, RapiMed Diagnostics, SA) to assess for proteinuria and leukocytosis, was performed in all patients. Treatment for TB was at the discretion of the attending physician.

### Tuberculosis Case Definitions

Based on clinical, radiological and microbiological criteria and the clinical impression of the attending physician, each patient was allocated to one of 4 diagnostic categories by a study investigator blinded to the LAM results. The reference standard was culture positivity for *M tuberculosis*. To avoid incorporation bias, the LAM ELISA results were not used to determine final TB status.

The categories were as follows:

1. **Definite TB**: A clinical presentation compatible with TB with at least 1 positive culture (from any specimen) for *M. tuberculosis* with response to anti-TB therapy [20].
2. **Probable TB**: A clinical-radiological picture highly suggestive of TB and/or anti-TB treatment initiated by the attending clinician based on clinical suspicion, but not meeting the above criteria for definite TB.
3. **Non-TB**: no evidence of TB based on smear microscopy and culture, and no radiological evidence to support the diagnosis of active TB, with or without an alternative diagnosis being established on patient follow-up.
4. **Indeterminate TB**: either the culture or CXR result (or both) were unavailable, and the patient was lost to follow-up or transferred to another centre, thus making it impossible to confidently rule-out or rule-in TB. These patients were excluded from the analysis.

Patients with TB and where relevant, non-TB cases, were followed up for at least 2 months to determine clinical response to therapy, and to confirm symptomatic recovery.

### LAM determination in urine and sputum

Mid-stream urine samples and sputum samples, collected in separate sterile containers from each patient at the clinic, were transported to the laboratory within 3 hours. A technician blinded to all clinical details and reference test results immediately processed the urine samples according to the manufacturer's instructions (Clearview TB® ELISA, Inverness Medical Innovations, U.S.A.). Briefly, an aliquot of each urine specimen was heated, cooled and then centrifuged, and the supernatants frozen at −80°C prior to batched ELISA testing. The sputum samples underwent liquefaction (addition of twice the volume of 0.1% DTT) prior to being processed and then frozen as for urine. Urine and sputum supernatants were thawed usually within 2–3 weeks of collection, and further processed according to the manufacturer's instructions. The plates were read immediately after processing at 450 nm on an ELISA plate reader (Anthos Labtec HT3). Duplicate positive and negative controls were performed with each run. Results were determined as the average of duplicate optical density (OD) readings for each sample minus [the average negative control OD reading plus 0.1], and using a cut-off of “zero” for positive and negative results.

### Detection of LAM in bacteria, other than *M. tuberculosis*, from sputum samples

Although not part of our original protocol, because early data showed poor specificity of sputum LAM (i.e. high positivity in non TB participants), we decided to investigate the likely cause by testing cultures and culture supernatants of mouth flora and specific organisms known to be mouth commensals e.g. aerobic Actinomycetes, which also contain LAM-like glycolipids in their cell walls. The organisms tested were isolated from the sputum samples of ∼15 non-TB patients. Multiple cultures were plated from these samples obtained from patients were not in any way matched to the study subjects. We therefore cultured a range of different microbes, including Nocardia and Streptomyces species (strain-typed where possible), *C.albicans*, *T. paurometabolum*, and *C. neoformans*. These microbes were all originally isolated from oral flora. Cultures were performed in both normal broth culture (containing yeast extract) and Todd-Hewitt culture media (without yeast extract). We also included normal oral flora from healthy controls containing mixed bacterial growth, predominantly viridans Streptococci. Microbial cultures were incubated at 37°C for 3–5 days, culture supernatants removed and the pellets resuspended in PBS. The LAM detection assay was performed as described above.

### Statistical Analysis

Sample size calculations were based on the assumption that 30% of 500 patients would have definite TB, and the resulting numbers would be sufficient to estimate the test sensitivity and specificity with a ±4% precision. Test accuracy results were computed as sensitivity, specificity and predictive values, along with 95% confidence intervals (CI). Stata IC, version 10 (Stata Corp, Texas, USA) was used for the logistic regression and Chi-squared analyses. In addition, multivariable, logistic regression analysis was conducted using culture-positive TB as the outcome to determine the incremental value of urine LAM over sputum smear alone and sputum LAM over both smear and urine LAM [21]. Receiver Operating Characteristic (ROC) curves of the logistic models were compared, where possible, to assess the overall accuracy and added value of both urine and sputum LAM [22], [23].

## Results

### Demographic and clinical characteristics

**Figure 1:**
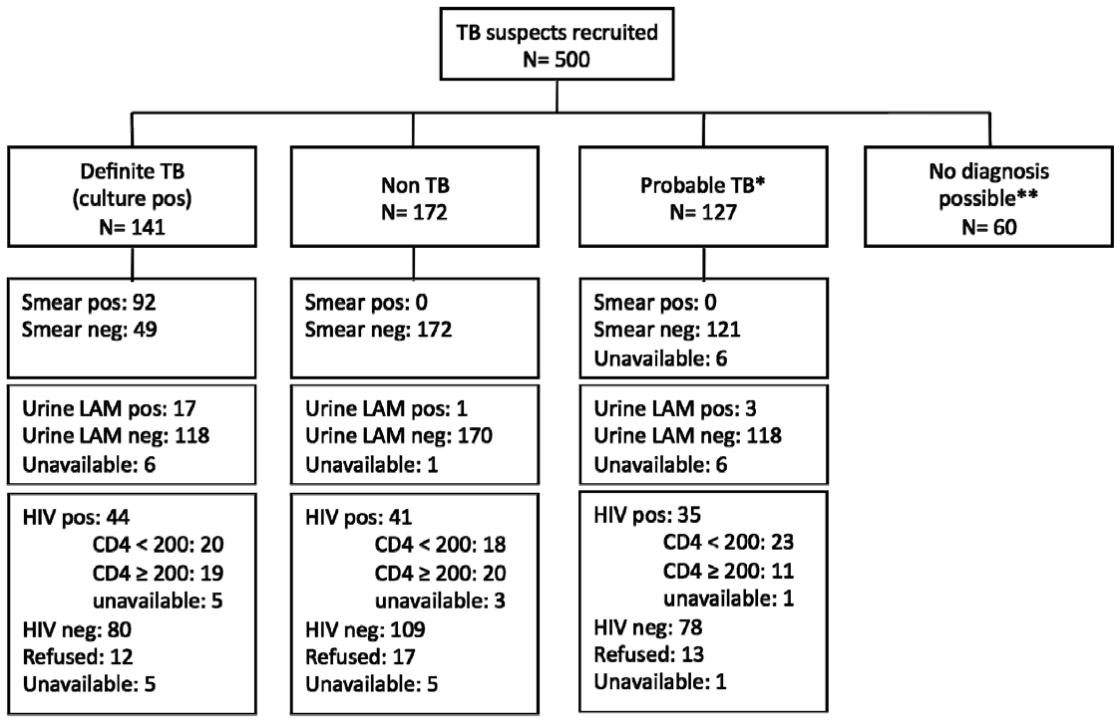
Flow chart of patients recruited to the study stratified by patient subgroup, smear microscopy, HIV status, and CD4 T cell count.

Sixty patients were excluded from further analysis because their TB status could not be reliably determined (see TB case definitions above), leaving 440 evaluable patients. Figure 1 shows the study recruitment flow chart including the patient numbers stratified by patient subgroups, smear microscopy, HIV status and CD4 T cell count. The demographic and clinical characteristics of the cohort, stratified by HIV status, are shown in Table 1. The predominant male and Black African population reflects the prevailing demography of TB in South Africa. The prevalence of HIV in the cohort was 31% and the median CD4 count in the HIV-infected population was 177 cells/mm^3^.

**Table 1:** Clinical and demographic characteristics of 440 evaluable TB suspects, stratified by HIV status.

	N (%)	HIV positive** (%)	HIV negative** (%)	P value
**Total TB suspects included**	440 (100)	120 (27.3)	267 (60.7)	
**Age, mean (SD)**	41 (12)	38 (10)	42 (13)	0.0006
**Sex**				
Male	291 (66.1)	61 (21)	195 (67)	
Female	149 (33.9)	59 (39.6)	72 (48.3)	<0.001
**Race**				
Black	314 (71.4)	97 (30.9)	178 (56.7)	
Mixed ancestry (coloured)	118 (26.8)	22 (18.6)	82 (69.5)	
White	8 (1.8)	1 (12.5)	7 (87.5)	0.015
**Weight in Kg, mean (SD) (n = 410)** *	61.2 (11.9)	58.3 (10.1)	62.0 (12.2)	0.0052
**Previous TB**				
Yes	161 (36.6)	46 (28.6)	101 (62.7)	
No	279 (63.4)	74 (26.5)	166 (59.5)	0.924
**Current Smoker (n = 437)** *				
Yes	252 (57.3)	55 (21.8)	170 (67.5)	
No	185 (42.0)	65 (35.1)	95 (51.4)	0.001
**Urine protein (n = 384)** *				
+	32 (7.3)	13 (40.6)	18 (56.3)	
++	15 (3.4)	4 (26.7)	10 (66.7)	
+++	3 (0.7)	0 (0)	3 (100)	
Negative	334 (75.9)	91 (27.2)	210 (62.9)	0.3624
**CD4 count, median (cells/mm^3^)(n = 111)** *	–	177	–	–

*Irreconcilable ambiguity on the request form precluding processing of sample, or patient consented but did not turn up for the test.

**Excludes 42 (9.5%) patients who refused HIV testing and 11 (2.5%) patients who had no data.

### Sensitivity of urine LAM versus smear microscopy in different categories of TB suspects

**Figure 2:**
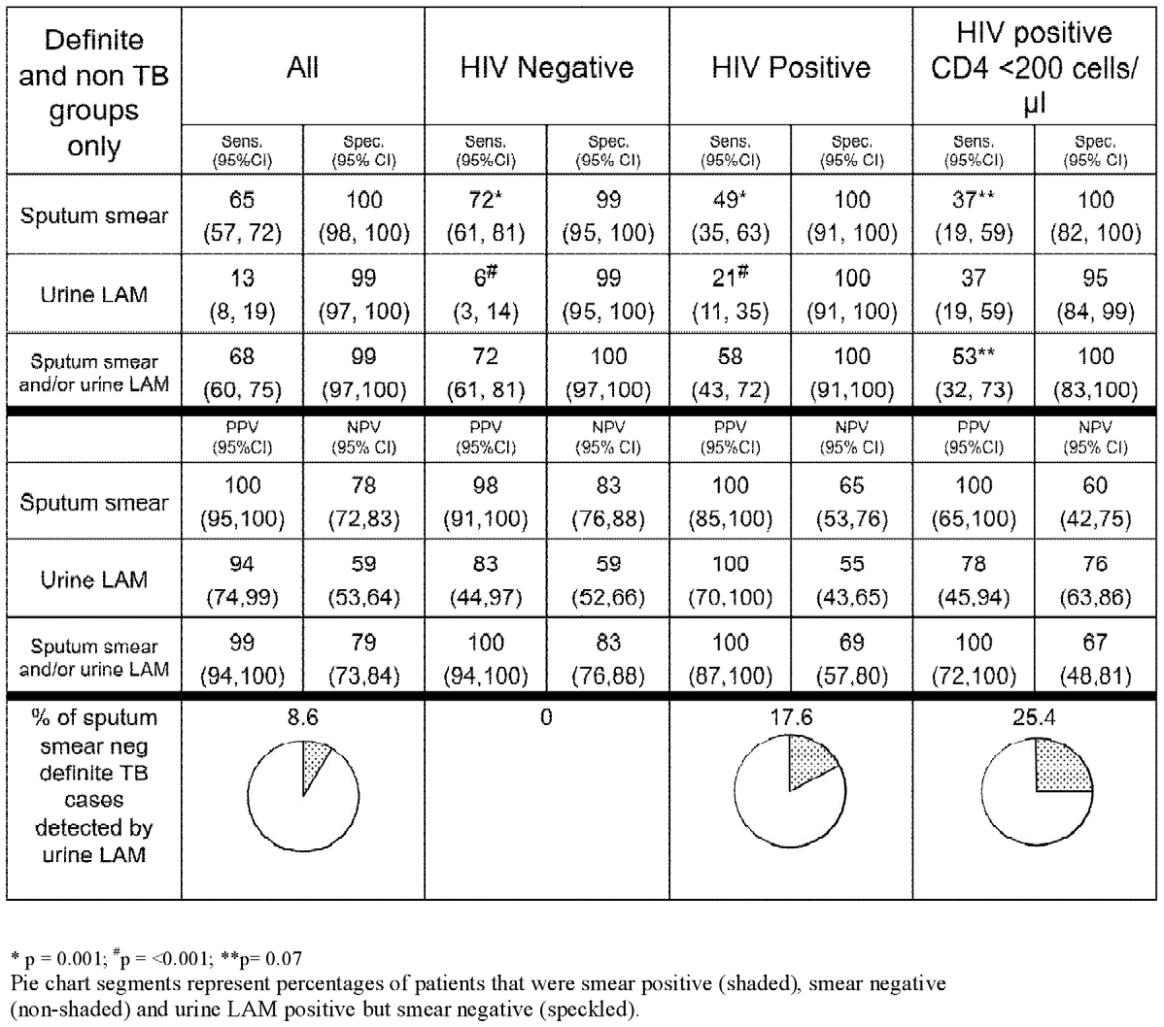
The sensitivity and specificity (and 95% confidence intervals (%); top panel of table) and positive and negative predictive value (middle panel of table) of smear-microscopy alone, urine LAM alone, and a combination of urine LAM and smear-microscopy. The bottom panel of the table shows the test sensitivity in smear negative culture positive TB patients. For sensitivity calculations the definite TB group (n = 141) was used whilst specificity calculations were performed using the non-TB group (n = 172) as a reference.

The sensitivity of urine LAM alone, smear microscopy alone, and urine LAM in combination with smear microscopy (using culture-positivity as a reference standard) is shown in Figure 2. Smear microscopy had a sensitivity of 65%, 49% and 37% in unselected TB patients, in HIV co-infected patients, and in those with a CD4 count <200 cells/mm^3^ respectively. By contrast, urine LAM had comparative sensitivities in the same groups of 13%, 21% and 37%, respectively. Thus, whilst the sensitivity of smear microscopy decreased significantly with increasing immune paresis (HIV-infected vs uninfected and CD4<200 vs >200) that of urine LAM increased significantly (Figure 2; p≤0.003). Smear microscopy was significantly more sensitive than urine LAM in all groups except TB patients with a CD4 count <200 cells/mm^3^, where sensitivities were equivalent. However, in this group with advanced HIV, the urine LAM assay and smear microscopy had non-redundant overlap i.e. the 2 tests tended to identify different patients. Thus, in TB patients with a CD4 count <200 cells/mm^3^ a combination of urine LAM and smear microscopy showed a sensitivity of 53% versus 37% for smear-microscopy alone; however, this additive value of urine LAM did not reach statistical significance (p = 0.07). Because of the smaller number of HIV-infected patients, comparative incremental multivariable ROC analyses could not be performed in this subgroup.

Collectively, these data indicate that 25% of HIV-infected smear-negative TB patients with a CD4 count <200 cells/mm^3^ had a positive urine LAM test (Figure 2). Moreover, in the smear negative/culture positive group, a significantly greater percentage of patients with CD4 count <200 cells/mm^3^ were urine LAM positive compared to those with CD4 count >200 cells/mm^3^ (p = 0.003; Figure 2). The same pattern was seen when the definite TB plus probable TB groups were combined for analysis (data not shown).

### Specificity of the urine LAM assay

The specificity of urine LAM and smear microscopy, using the non-TB group as a reference, in the different patient subgroups is shown in Figure 2. In summary, LAM was highly specific (95 to 100%) in all subgroups but specificity was comparatively lowest in the CD4<200 cells/mm^3^ subgroup (but not statistically different from other subgroups). Thus urine LAM is a reasonable rule-in test for active TB in those with a CD4 count <200 cells/mm^3^ (sensitivity  = 37% and specificity  = 95%).

### Association of urine LAM with HIV status and urine protein

Urine LAM positivity was significantly higher in HIV-infected vs uninfected subjects (21% vs 6%; p<0.001; Figure 2) and those with a CD4 count <200 cells/mm^3^ vs >200 cells/mm^3^ (37% vs 0%; p = 0.003; Figure 2). In the univariate analysis a positive urine LAM result was associated with HIV positivity (OR = 4.75; p = 0.002), but inversely associated with low weight (OR = 0.94; P = 0.01), and previous TB (OR = 0.09; p = 0.02). However, in the multivariable analysis LAM positivity was independently associated only with HIV positive status (OR = 3.2; P = 0.04) and lack of previous TB (OR = 0.09; P = 0.02).

In HIV-infected culture positive TB patients (n = 36) there was no association between urine LAM and proteinuria measured through urine dipstix readings i.e there was no significant difference between the proportion of patients who were urine LAM positive in those who did and did not have proteinuria on dipstick examination [6/24 (25%) vs 2/12 (17%)].

### Sputum LAM performance outcomes

**Figure 3:**
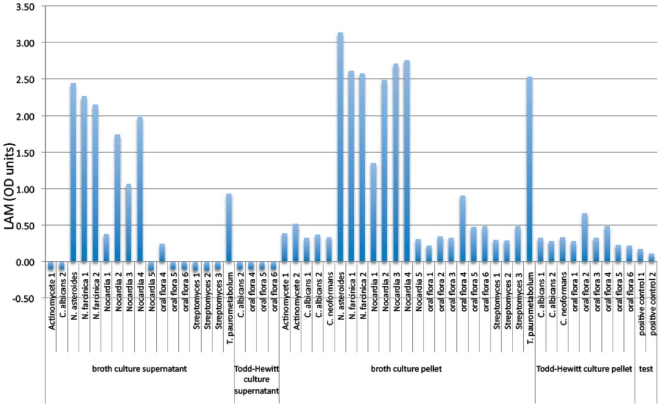
LAM positivity (> zero OD units =  positive for LAM after subtraction of the negative control i.e. cutpoint is zero) in cultures of oral mouth flora (oral flora) and in organism-specific cultures (various Actinobacteria, including different strains of Nocardia and Streptomyces, and *C. albicans*, *T. paurometabolum*, and *C. neoformans* inoculated into normal broth culture [containing yeast extract] and Todd-Hewitt culture media [without yeast extract]. Normal oral flora from six different healthy control subjects was also cultured).

Of the 440 evaluable patients sputum samples were available for LAM analysis in 377 patients. Compared to urine LAM, sputum LAM had a higher sensitivity (86%; 95%CI 81, 90%) but poorer specificity (15%; 95% CI 10, 21%; Table 2). Cultures of oral flora from non-TB patients and of specific microbes known to reside in the oral cavity tested positive for LAM using the ELISA kit (Figure 3). In the multivariable analysis, sputum LAM did not add significantly to the overall accuracy beyond urine LAM and smear microscopy (area under the ROC of 85.15% vs 83.96%, p = 0.14) [ROC curves not shown].

**Table 2:** Measures of accuracy for sputum LAM in definite TB patients, and in definite plus probable TB patients, stratified by HIV status and CD4 count.

		Sensitivity (95% CI)	Specificity (95% CI)	PPV (95% CI)	NPV (95% CI)
**Definite TB (culture pos)** (Non-TB group used for specificity calculations)	All patients	86 (81, 90)	15 (10, 21)	60 (54, 65)	42 (30, 56)
	HIV neg patients	**84** * (74, 91)	16 (10, 24)	40 (32, 48)	60 (41, 77)
	HIV pos patients	**100** * (90, 100)	7 (2, 19)	46 (35, 57)	100 (44, 100)
	CD4 <200	**94** # (72, 99)	50 (33, 67)	50 (33, 67)	94 (72, 99)
	CD4 ≥200	**100** # (82, 100)	17 (6, 39)	53 (36, 69)	100 (44, 100)
**Definite TB and Probable TB** (Non-TB group used for specificity calculations)	All patients	86 (81, 90)	15 (10, 21)	60 (54, 65)	42 (30, 56)
	HIV neg patients	**82** ** (75, 88)	16 (10, 24)	57 (50, 64)	39 (26, 55)
	HIV pos patients	**92** ** (82, 96)	7 (2, 19)	59 (48, 68)	38 (14, 69)

*p<0.0001 (comparison of sputum LAM sensitivity in HIV positive vs HIV negative definite TB cases).

# p = 0.0131 (comparison of sputum LAM sensitivity in HIV positive subjects with a CD4 count ≥200 vs CD4 count <200 cells/mm^3^).

**p = 0.04 (comparison of sputum LAM sensitivity in HIV positive vs HIV negative definite plus probable TB cases).

## Discussion

In this study we show that in unselected HIV-infected or uninfected ambulant TB suspects LAM has little clinical utility in a high burden primary care setting (poor sensitivity of only 13% and 21%, respectively in these subgroups). However, in the diagnostically challenging smear negative/culture positive HIV-infected subgroup with a CD4 count <200 cells/mm^3^, 25% of patients (n = 19) had a positive urine LAM. The high specificty of the assay thus makes urine LAM a potential rule-in test for active TB within this sub-group. At first glance the sensitivity of 25% in this subgroup appears low, but given the high specificity of the assay, its modest cost, generation of results within 24 hours (potentially even faster, if a LAM strip test can be commercialized), the lack of other alternative rapid diagnostic options, the diagnostic conundrum presented by these cases [5], and that a point-of-care (POC) test format is currently being validated, makes urine LAM a potentially useful assay. Moreover, other established TB tests such as nucleic acid amplification tests have a sensitivity (∼30%) within a similar range, and a high accompanying specificty when used clinically as rule-in tests for extra-pulmonary TB [24].

In this study, we have attempted to define the specific utility of urine LAM within a primary care population with a high prevalence of HIV. Other investigators have found that urine LAM sensitivity in smear-negative TB, using the pre-commercial version, varied between 28% to 56% [15], [17], [18]. However, urine LAM outcomes stratified by CD4 count were not available in two of the studies [16], [17], and specificity was suboptimal in both of them (discussed later). Thus, there are no existing data about urine LAM outcomes in specific CD4 categories in patients who present to primary care clinics with suspected TB. This information is crucial because the vast majority of TB suspects from resource-poor settings present to primary care facilities. If additional studies confirm our findings, there is potential to develop an algorithm where patients who are smear negative, HIV positive and have a CD4 count <200 cells per mm^3^ may be ideal candidates for the urine LAM assay (especially the POC version). It is this very population that poses a diagnostic conundrum for clinicians because of the atypical radiological picture and the high proportion of smear negativity. Given the wide-spread availability of rapid HIV testing, and in the near future, POC CD4 tests, we believe that urine LAM could easily be incorporated into a diagnostic algorithm for TB. Thus, our work has identified a specific subgroup that should now be targeted in future studies so that the utility of the urine LAM assay in HIV-infected subjects can be further clarified. Future studies should also evaluate the efficiacy and cost-effectiveness of an primary care algorithm-based approach using urine LAM.

The specificity of the urine LAM assay was high, although it was lower in those with a CD4 count, 200 cells/ml, and similar to studies done in hospitalised patients with suspected TB [18] and in individuals being screened for TB prior to antiretroviral therapy [15]. This contrasts with the poorer specificity of approximately 88% in three other studies [16], [17], [19]. This may possibly be explained by undiagnosed occult TB disease and may also explain the lower specificty in our study in the most immunocomprimised patients. Other reasons may include possible detection of antigen from latently-infected subjects, contamination of the sample by environmental pathogens or rapidly dividing bacteria (particularly if the sample was collected at home and brought to the clinic), or contamination by non-bacterial species e.g. *Candida*, which is prevalent in HIV-infected populations. These possibilities are tenable given our findings that several organisms, including Candida species, cross-react with the polyclonal LAM antibodies within the assay. In our study urine was collected at the clinic in sterile containers; however, the collection methods used in the other studies were not described.

The urine Clearview® LAM ELISA used in this study is the current commercially-available version, and supersedes the prior pre-commercial prototype (MTB LAM ELISA; Chemogen, Portland, USA). Although the polyclonal antibody used in the Clearview® LAM ELISA is the same as in the pre-commercial version, there are differences in the manufacturing technology and protocols used (e.g. antibody coating technologies used etc). Whether this may impact test performance, and make the different versions incomparable, seems unlikely but remains a possibility given that several technical factors may impact on ELISA test performance [25].

Our test sensitivity of 21% is much lower than the sensitivity of 61% found by Reither *et al* in ambulant HIV-infected Tanzanian TB suspects using the Chemogen MTB ELISA [17], 52% by Mutetwa *et al* in HIV-infected ambulant Zimbabwean TB suspects using the same prototype [16], and 38% found by Lawn *et al* in ambulant South African HIV-infected patients being screened prior to the initiation of HAART also using the pre-commercial prototype [15]. How do we explain these inconsistent findings? Our patients were less immuno-suppressed (median CD 4 count of  = 177) than other cohorts [15], [17], [18]; there appears to be a crude inverse relationship between CD4 T cells count and urine LAM sensitivity [15], [17], [18]. Furthermore, ambulant patients in our study may have presented earlier with less advanced disease given the better health care infrastructure in South Africa compared with Tanzania. Other factors such as strain type, population genetics and malnutrition, though unproven, may also have played a role. Another possibility is population-specific heterogeneity [26] and differential HIV-related glomerular podocyte dysfunction, which determines the ‘leakiness’ of the renal glomerulus for proteins [27]. However, the lack of association between urine LAM and proteinuria in our study, in contrast to that of Reither et al [17], argues against this hypothesis.

The standardized Clearview® LAM ELISA has not previously been evaluated using sputum samples. We hypothesised, based on a prior study of LAM antigen in sputum [28], that detection of LAM antigen in smear negative patients would be diagnostically useful. However, although the Clearview® TB ELISA had a high sensitivity in sputum samples, the specificity was dismally poor. We determined that this was probably due to cross-reactivity with LAM-like microbial carbohydrate surface molecules in the cell walls of several mouth-residing organisms such as Candida, and many species of Actinobacteria. Colonisation by Candida, or else mucosal or systemic candidiasis (e.g. mouth and genitals), may also possibly explain the lower specificity of the LAM assay seen in high HIV prevalence populations in other settings [16], [17], [19]. Certainly the polyclonal antibodies contained within the test, despite not cross-reacting with common pathogens like *E. coli, S. pneumoniae* etc [10], are not specific for mycobacterial species.

A limitation of our study is the low number of HIV-infected definite TB cases who were smear negative and had a CD4 count less than 200 cells/mm^3^ (n = 19), and the resulting wide confidence intervals in this subgroup. Given that our study was not adequately powered to evaluate outcomes in HIV-infected individuals with advanced immunosuppression our findings can only be regarded as preliminary. Thus, even in a high HIV prevalence setting, large and adequately powered studies will be required to recruit sufficient patients with smear negative definite TB in the low CD4 count subgroup to achieve reliable estimates of performance outcomes. Although we found that urine LAM plus smear microscopy may be more sensitive than smear microscopy alone in those with low CD4 counts, this was not statistically significant (p = 0.07). This is likely due to type II error and the low sample numbers in the low CD4 subgroup. Larger and adequately powered studies targeting this particular subgroup are now required to clarify utility.

In conclusion, LAM is not a useful assay in unselected TB suspects in a primary care setting. However, a targeted approach to using LAM may be useful in those who have advanced HIV infection, though, even in this group sensitivity is modest. We have interpreted this positively given the prevailing diagnostic challenges and lack of other diagnostic options within this subgroup. Our findings, despite the limited assay sensitivity, may lend themselves to useful application in diagnostic algorithms, incorporating CD4 count and smear status, within high burden countries. Larger targeted studies, in the low CD4 count subgroup, in ambulant and hospitalised TB populations from different geographical settings are now required to validate this approach and clarify our findings.
